# Evaluation of an Elastodontic Bioactivator Versus High-Pull Headgear for the Treatment of Skeletal Class II Hyperdivergent Pediatric Patients: A Retrospective Study

**DOI:** 10.3390/jcm15020804

**Published:** 2026-01-19

**Authors:** Maria Francesca Sfondrini, Maurizio Pascadopoli, Maria Gloria Nardi, Filippo Cardarelli, Paolo Zampetti, Annalisa Viola, Suzanna Labadze, Andrea Scribante

**Affiliations:** 1 Unit of Orthodontics and Pediatric Dentistry, Section of Dentistry, Department of Clinical, Surgical, Diagnostic and Pediatric Sciences, University of Pavia, 27100 Pavia, Italy; 2 Department of Interdisciplinary Medicine, School of Medicine, University of Bari “Aldo Moro”, 70124 Bari, Italy

**Keywords:** high-pull headgear, elastodontic devices, hyperdivergence, class II, malocclusion

## Abstract

**Background/Objectives**: This retrospective study evaluated and compared the cephalometric effects of an elastodontic bioactivator and conventional high-pull headgear in growing patients with hyperdivergent Class II malocclusion. **Methods**: Patients aged 7–11 years were divided into two groups according to the appliance used for the orthodontic treatment performed: elastodontic device (ED) and high-pull headgear (HPHD). Cephalometric measurements were recorded at baseline (T0) and after 18 months of treatment (T1). The data were subjected to statistical analysis, descriptive statistics were calculated, and an ANOVA test and post hoc Tukey test were performed (repeated measures correction was applied for intragroup comparisons). Linear regressions were conducted. Significance was predetermined as *p* < 0.05 for all the tests performed. **Results**: 40 patients were included, 20 belonging to the ED group and 20 to the HPHD group. Both groups showed a significant increase in SNB (*p* < 0.05), suggesting favorable mandibular positional changes. SNA and ANB did not show significant intra- or intergroup variations (*p* > 0.05). Regarding vertical skeletal parameters, no significant intra- or intergroup changes were observed at T0 and T1, indicating that both devices preserved vertical stability without worsening the hyperdivergent pattern. Dentoalveolar and soft-tissue effects were limited. **Conclusions**: Both ED and HPHD are effective in managing hyperdivergent Class II growing patients. The two appliances provide comparable improvements in mandibular positioning. Both devices seem to preserve vertical skeletal dimensions, avoiding further mandibular clockwise rotation. Both appliances are associated with minimal undesirable effects on the soft tissues.

## 1. Introduction

Class II orthodontic malocclusions are very common and can be treated with a great variety of appliances [1,2]. However, these malocclusions have been shown to be often associated with hyperdivergent growth [3]. Hyperdivergent patients with class II malocclusion are characterized by a mandibular clockwise rotation, resulting in an increased occlusal plane angle, a convex profile, and a mandibular retrognathia [4,5]. It is important to control hyperdivergence in growing patients to achieve satisfying outcomes [6,7]. Different strategies have been proposed to treat hyperdivergent subjects with class II malocclusion. Traditional extra-oral appliances, such as headgear, offer excellent anchorage control, skeletal modification, and the ability to precisely modulate the magnitude and direction of force vectors, providing a customized appliance for each individual patient [8]. These devices are highly dependent on patient compliance, and their clinical effectiveness correlates strongly with wear time [9]. Furthermore, these appliances may be invasive in terms of social acceptability and comfort, as their external visibility can negatively impact patient compliance and psychosocial well-being [10]. Moreover, rare but severe complications have been reported, including ocular injuries and even blindness caused by accidental release of the facebow or detachment of the elastic modules [11]. Additional risks include soft-tissue trauma, facial scarring, and dental or skeletal side effects such as undesired buccal tipping of molars [12]. For these reasons, careful instruction, safety modules, and patient monitoring are essential when prescribing this device.

Recent novelties in interceptive orthodontic therapy increasingly include preformed intra oral appliances, which are not custom made but rather manufactured in standardized sizes [13,14]. Numerous appliances have been studied and used for the treatment of malocclusions and the correction of bad oral habits and atypical swallowing, such as Froggy mouth, Habit Corrector, Face Former, and elastodontic appliances [15]. Advantages of these devices include their simplicity of use, low cost, and reduced chair time, since no custom fabrication is required [14]. Some clinical studies suggest positive outcomes in terms of arch development, correction of functional disorders, and improvements in dentoalveolar relationships in growing patients [13,16]. However, these appliances present several limitations: these devices are available in standard sizes (e.g., small, medium, large) and may not perfectly adapt to each patient’s craniofacial anatomy, potentially reducing biomechanical precision and long-term stability [17]. Their clinical effectiveness is also highly dependent on patient compliance, and poor adherence may compromise treatment results [13]. Some mechanical limitations have been also reported, such as deformation under functional loading, which may decrease the predictability of clinical outcomes [18]. According to Di Vecchio et al., these appliances are generally suitable only for mild to moderate functional discrepancies and early interceptive purposes, whereas severe malocclusions often require conventional or custom-made approaches [17].

Elastodontics represent an innovative treatment option: this term refers to a recent technique involving removable, preformed, silicone elastomer appliances exerting small, elastic, and biological forces [18,19,20]. These devices represent a simple and more comfortable option to correct malocclusions, favorably modifying growth at developmental age [21]. Elastodontic appliances are proposed for orthopedic effects, allowing teeth to find their position due to the absence of indentations. These appliances are also able to properly rehabilitate muscular and tongue functions [22].

Elastodontic devices are also proposed for the treatment of hyperdivergent class II malocclusions, since they promote mandibular advancement and guide teeth eruption to control vertical discrepancies [23]. The aim of the present study was to compare sagittal and vertical cephalometric outcomes and the dentoalveolar and soft tissue effects of a conventional interceptive treatment performed with high-pull headgear (HPHD) versus an elastodontic device (ED) for the treatment of hyperdivergent Class II growing patients. The first null hypothesis is that there are no significant differences in vertical skeletal parameters between the two treatment groups. The second null hypothesis is that there are no significant differences in sagittal skeletal parameters between patients treated with an ED and patients treated with HPHD. The third null hypothesis is that there are no significant differences in dentoalveolar or soft tissue parameters between the two groups after treatment.

## 2. Materials and Methods

### 2.1. Trial Design

This was an observational retrospective study approved by the Unit Internal Review Board (registration number: 2024-0110) and registered on Clinicaltrials.gov (NCT number: NCT06281613).

### 2.2. Participants

This study was conducted on patients who completed their orthodontic treatment at the Unit of Orthodontics and Paediatric Dentistry, Section of Dentistry, Department of Clinical, Surgical, Diagnostic and Paediatric Sciences of the University of Pavia (Pavia, Italy). The inclusion and exclusion criteria adopted for the enrollment of patients are shown in Table 1.

### 2.3. Interventions and Outcomes

Each patient routinely underwent an orthodontic evaluation, including dental arch impressions, intraoral and extraoral photographs, orthopantomography, and lateral cephalometric radiographs according to Good Clinical Practice. All cephalograms were taken with the same radiologic unit (Soredex Cranex-D Digital, Orion Corporation, Helsinki, Finland). Cephalometric tracing was performed with Delta-Dent software (Delta-Dent version 1.7, Outside Format, Spino d’Adda (CR), Italy) following Giannì cephalometric analysis. All cephalometric parameters considered are shown in Table 2.

**Figure 1:**
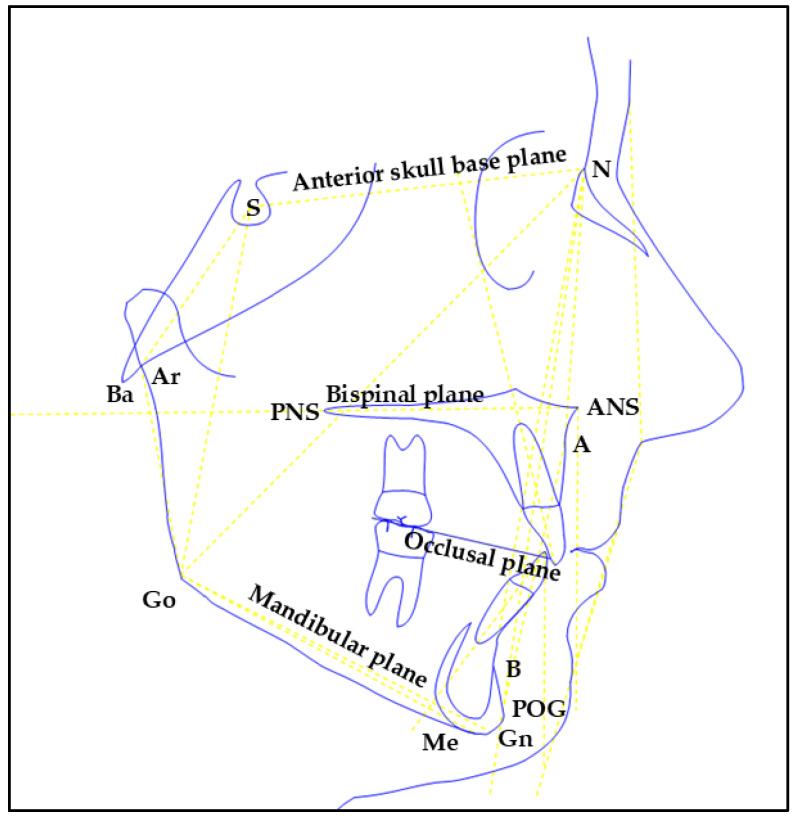
Simplified sagittal schematic of the main cephalometric landmarks and planes.

The main cephalometric landmarks and planes considered in this study are schematically illustrated in Figure 1.

**Figure 2:**
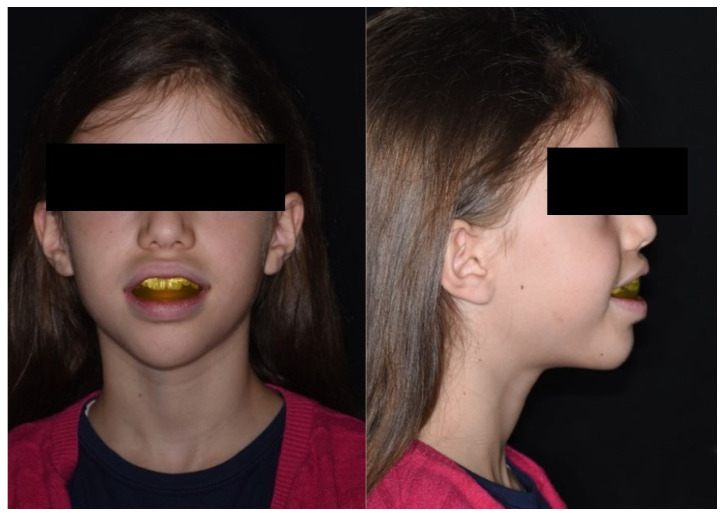
Extraoral photographs of a patient wearing an elastodontic device (ED).

**Figure 3:**
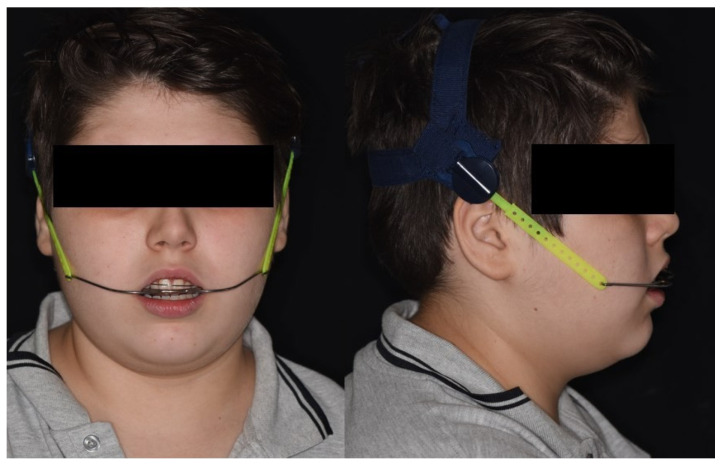
Extraoral photographs of a patient wearing high-pull headgear (HPHD).

The cervical vertebral maturation (CVM) method was applied to determine the craniofacial skeletal maturational stage of the enrolled patients [24]. After orthodontic evaluation, a treatment plan was proposed to the parents. After acceptance and a signature of informed consent given by the patients’ parents, orthodontic devices were delivered to the patients, who received detailed information about their proper use (T0). Patients were enrolled if they underwent an orthodontic treatment with one of the following appliances:

- ED, elastodontic device (AMCOP® Integral; O.P. AMCOP S.r.l., Capurso (BA), Italy; patented by Micerium S.p.A) (Figure 2): AMCOP Integral is a prefabricated, monobloc, elastodontic appliance manufactured from a thermo-activable polymer/elastomer blend. This bio-activator incorporates vestibular and lingual soft-tissue shields, which delimit a central free area for the teeth, enhancing neuromuscular forces while minimizing perioral interference. The Integral variant specifically features a flat occlusal plane and is supplied in arch forms (S, ØS, F, C) to match craniofacial and arch morphology; color-coded variants (e.g., blue, orange, red) with distinct mechanical responses have been evaluated in vitro [23,25]. This appliance aims to achieve orthopaedic dentoalveolar rebalancing, including correction of altered occlusal curves, management of anterior/lateral open bite, and coordination of the arches. This device also provides orofacial muscle and tongue posture re-education [22]. ED is manufactured in different sizes; for each patient, the proper size of the ED was selected by clinically assessing the transverse width of the dental arches and verifying intraorally that the device properly matched the patient’s arch form and ensured adequate stability [22]. Patients were asked to wear the orthodontic device during the night and one hour per day. They were informed that they could not speak while wearing the device and that it was normal to feel anterior teeth discomfort at first, especially in the lower arch.

- HPHD, high-pull headgear (Bio-High-Pull Headgear; FORESTADENT Bernhard Förster GmbH, Pforzheim, Germany) (Figure 3): HPHD is an extraoral orthopaedic device consisting of bilateral facebows connected to maxillary molar bands and an occipital strap that delivers upward and backward orthopaedic forces. The outer bow transmits the elastic tension from the headcap to the inner bow, which is inserted into the molar tubes; by adjusting force magnitude and direction, the appliance applies a posteriorly directed vector with an upward component [26,27]. This appliance aims to restrict forward and downward maxillary growth and to distalize and intrude maxillary molars, thereby controlling the vertical dimension and reducing the tendency toward posterior mandibular rotation [27,28]. Orthodontic bands (*3M™ Victory Series™ First Molar Bands*; micro-etched, narrow/standard contoured; 3M (Solventum, formerly 3M Oral Care/3M Unitek), Monrovia, CA, USA) were cemented to the upper first permanent molars using a glass ionomer band cement (*3M™ Unitek™ Multi-Cure Glass Ionomer Band Cement*; 3M Company, St. Paul, MN, USA), and cement was light-cured using a light-curing unit (*Starlight Pro*; power output 1400 mW/cm^2^; Mectron S.p.A., Carasco, Italy). HPHD was applied, and patients were asked to wear the device 14 h per day and taught to safely wear and remove while it avoiding injury.

After 18 months (T1), updated intraoral and extraoral photographs and lateral cephalometric radiographs were taken to detect, assess, and compare the cephalometric outcomes resulting from the two differently treated groups. All cephalometric measurements were performed by the same operator. Intra-rater reliability was calculated, resulting in an intraclass correlation coefficient (ICC) of 0.99.

### 2.4. Sample Size

The sample size for two independent study groups and a continuous primary endpoint was calculated using the ClinCalc.com tool (accessed on 17 December 2025) [29], setting alpha = 0.05 and power = 80%. The variable SN^GoGn was chosen as the primary outcome. An expected mean of 33.9 and an expected mean difference of 3.9 with a standard deviation of 4.39 were hypothesized based on results from previous literature [30]. With these premises, 40 patients were required for the study, equally divided into the two study groups. No dropout rate was recorded.

### 2.5. Statistical Analysis

Data underwent statistical analysis with R Software (R version 3.1.3, R Development Core Team, R Foundation for Statistical Computing, Wien, Austria). Descriptive statistics (mean, standard deviation and effect size) were calculated for each group and variable. Data normality was assessed using the Kolmogorov–Smirnov test. Baseline between-group comparisons were performed using the Mann–Whitney U test due to the non-normal distribution of baseline demographic variables. For longitudinal analyses, the data met normality assumptions; therefore, an ANOVA followed by Tukey’s post hoc tests was applied (repeated measures correction was applied for intragroup comparisons). Effect sizes were calculated using Cohen’s d to quantify the magnitude of intra-group changes over time. Effect sizes were interpreted as small (d = 0.20–0.49), moderate (d = 0.50–0.79), or large (d ≥ 0.80). A Bonferroni correction for multiple results was applied to adjust the significance level for cephalometric measures and univariate linear regressions, multiplying *p* values for the number of multiple comparisons. Linear regressions were conducted to evaluate the effects of different variables. Significance was predetermined as *p* < 0.05 for all the tests performed.

## 3. Results

### 3.1. Participant Flow and Baseline Data

**Figure 4:**
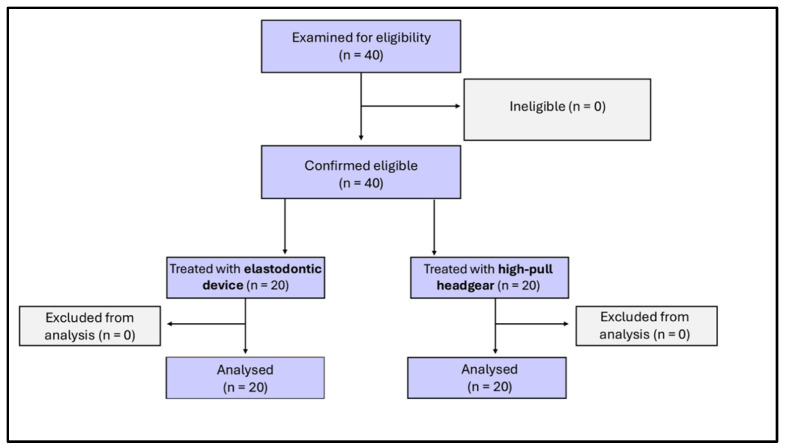
Flow chart of the study population.

Fourty patients were enrolled according to the inclusion criteria; they agreed to participate and received the allocated interventions. No patients were excluded from the analysis. The flow chart of the study is shown in Figure 4.

At the baseline (T0), the sample included two groups of patients:

- A trial group consisting of 20 patients (11 males and 9 females) with a mean age of 9.2 ± 1.2 years treated with ED;
- A control group consisting of 20 patients (7 males and 13 females) with a mean age of 9.6 ± 1.7 years treated with HPHD.

A Mann–Whitney U test confirmed the absence of significant differences in baseline demographic and maturational characteristics between groups (*p* > 0.05).

The characteristics of the study sample are shown in Table 3.

### 3.2. Cephalometric Outcomes

The cephalometric parameter scores are presented in Table 4. To show inter- and intragroup differences, a letter-based comparisons system was used. Letters were assigned to means so that same letter/letters between groups denotes that no significant difference exists [31].

Considering SNB scores, intragroup comparisons showed significant differences in the intervals T0–T1 in both groups (*p* < 0.05). As far as S-N^Go-Gn, AnsPns^Go-Gn, and N-Go-Gn scores are concerned, a significant difference was observed between the control group at T0 and the trial group at T1 (*p* < 0.05). No other intra- or intergroup significant differences occurred (*p* > 0.05).

Effect size analysis supported the statistical findings. Moderate to large effect sizes were observed for SNB in both treatment groups, indicating clinically meaningful mandibular positional changes. In contrast, vertical and dentoalveolar parameters generally showed small or negligible effect sizes, suggesting limited clinical impact.

**Figure 5:**
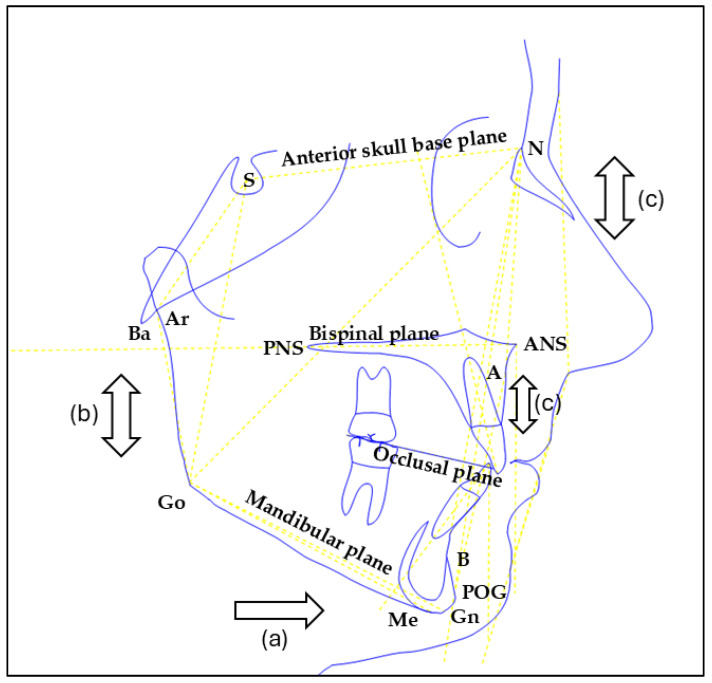
Conceptual schematic representation of treatment-related sagittal (a), vertical (b), soft tissue, and dentoalveolar changes (c) observed in the ED and HPHD groups.

To facilitate interpretation of the main findings, a conceptual schematic representation of treatment-related sagittal, vertical, soft tissue, and dentoalveolar changes observed in the ED and HPHD groups is provided in Figure 5.

### 3.3. Linear Regression Analysis

Linear regressions showed that cephalometric scores were not significantly influenced by the age and cervical vertebral maturation stage of the patients (*p* > 0.05).

## 4. Discussion

The present study evaluated the cephalometric effects of an ED versus a conventional HPHD in growing patients with hyperdivergent Class II malocclusion. The main findings were that both appliances were associated with a significant intragroup increase in SNB, whereas SNA and ANB did not show significant differences at the end of the treatment. Regarding vertical skeletal parameters, no significant intra- or intergroup changes were observed from T0 to T1 in either the control or the trial group. Dentoalveolar and soft-tissue changes were not relevant either group. The inclusion of effect size analysis allowed for a more clinically oriented interpretation of the results, highlighting that statistically significant findings were also associated with moderate to large magnitudes of effect, particularly for sagittal mandibular changes, whereas most non-significant outcomes were characterized by negligible effect sizes. The first null hypothesis was partially rejected. In the present study, both ED and HPHD protocols were associated with a statistically significant increase in SNB, suggesting a favorable mandibular positional change over the observation period. This finding aligns with previous studies on elastodontic appliances, which documented significant SNB increments, generally ranging from 0.8° to 1.5°, reflecting enhanced mandibular growth or forward positioning through neuromuscular adaptation [32]. Cardarelli et al. also observed a positive change in SNB with ED treatment, comparable in magnitude to functional appliances such as the Fränkel-2 [32]. Conversely, previous studies on HPHD reported either a slight increase or maintenance of SNB, with the sagittal improvement often attributed to reduced maxillary forward displacement rather than direct mandibular enhancement [33,34]. The similarity in SNB increase between HPHD and ED in the present study suggests that, despite differing biomechanical actions—neuromuscular stimulation versus orthopaedic restraint—both appliances can achieve a comparable short-term mandibular positional benefit in growing hyperdivergent Class II patients. It should be noted that patients enrolled in this study were aged between 7 and 11 years old; according to Franchi et al., in females, the mean age immediately preceding the mandibular growth spurt is estimated at 12.1 ± 1.1 years, whereas in males, it is delayed to approximately 13.2 ± 0.8 years [35]. Therefore, mandibular growth may have played a key role in the increase in SNB value and improvement of malocclusion, especially in females, even if no significant differences related to sex occurred. However, previous studies show that despite the potential for mandibular growth, it is unlikely that complete self-correction of malocclusion will occur without adequate treatment [36,37]. SNA values did not significantly vary in the HPHD and ED groups, indicating that neither protocol produced significant maxillary anterior displacement or restriction during the observation period. This is in accordance with other studies in the literature that demonstrated minimal effects of elastodontic appliances on SNA due to their primary action on mandibular advancement rather than maxillary restraint [22,23]. On the contrary, several studies reported that HPHD induces a modest but significant reduction in SNA—typically around 1°—through orthopedic limitation of maxillary forward growth [38]. The absence of SNA reduction in the headgear group of the present study may reflect differences in patient age, compliance, treatment duration, or baseline growth pattern compared to these earlier investigations. In both groups, ANB remained unchanged despite the increase in SNB due to the absence of significant SNA reduction. This finding contrasts with several studies on functional appliances or HPHD, where sagittal improvement was reflected in both increased SNB and decreased SNA, leading to significant ANB reduction [28,39]. The lack of ANB significant variation in the present study suggests that sagittal correction was modest and mainly related to mandible positional change and growth. The second null hypothesis was accepted. Concerning vertical skeletal changes, both HPDHD and ED groups maintained stable mandibular angle values, indicating vertical stability without exacerbation of the hyperdivergent pattern. Conversely, other studies in the literature reported a significant decrease in mandibular plane angle following HPHD therapy in growing Class II patients, with reductions typically ranging from 1° to 2° [33], supporting HPHD vertical control effect through inhibition of maxillary molar extrusion and upward–forward rotation of the mandible [26]. Similarly, Tulloch et al. documented vertical control with headgear, noting a downward trend in S-N^Go-Gn compared to untreated controls [34]. Grögli et al. observed in their retrospective cohort study that positive vertical effects achieved by HPHD were stable over time [40]. However, other studies compared the effects of cervical headgear and HPHD on vertical dimensions in Class II growing patients, and cervical headgear showed more control over the vertical dimension [41,42]. Nonetheless, further studies disproved the effectiveness of HPHD in vertical controlling [43]. Concerning elastodontic appliances, many studies showed no significant variations in mandibular angle after treatments. These devices appear to preserve the vertical dimension, likely due to their light continuous forces, absence of vertical extrusion mechanics, and neuromuscular re-education effects [20,21]. Cardarelli et al. also reported stable vertical parameters in children treated with various elastodontic protocols, supporting the present findings [32]. Vertical stability observed with both HPHD and ED may be advantageous in hyperdivergent patients where further mandibular clockwise rotation is undesirable. Conversely, when reduction of mandibular plane angle is a primary treatment goal, neither ED nor HPHD appears to represent a fully predictable treatment option. The third null hypothesis was accepted, since dentoalveolar and soft-tissue measures were stable within groups over the study period. The clinical implication is that both protocols achieved skeletal control with minimal unwanted soft-tissue effects. In agreement with our findings, other studies demonstrated that functional therapies generally produce modest dentoalveolar adjustments with limited soft-tissue impact. A recent systematic review and meta-analysis focused on soft-tissue outcomes in Class II growing patients reported small favorable changes—upper-lip retraction relative to the E-line and an increase in nasolabial angle—while overall profile modifications remained limited [44]. The present study has some limitations. Firstly, the two treatments differed in prescribed wear time (ED: night-time plus 1 h/day; HPHD: ~14 h/day); although wear time prescriptions differed between ED and HPHD in accordance with clinical recommendations [32,45], patient adherence was not objectively evaluated. As a result, differential compliance may have contributed to the observed intergroup differences and should be considered when interpreting comparative effectiveness. Furthermore, it would be necessary to assess the stability of results obtained over time. This study design was not randomized, and although baseline characteristics were comparable between groups, potential selection bias cannot be entirely excluded. Moreover, the assessment relied exclusively on two-dimensional cephalometric analysis, which may not fully capture three-dimensional skeletal and soft tissue adaptations. Moreover, although baseline equivalence between groups was confirmed through formal statistical testing, a small proportion of patients at CVMS 3 was present only in the HPHD group. Therefore, growth-related confounding factors cannot be entirely excluded. In addition, linear regression analyses assessing the influence of age and cervical vertebral maturation stage may have been underpowered given the relatively small sample size. Consequently, conclusions regarding the absence of maturation-related effects should be interpreted with caution. Future studies are needed to assess the stability of effects over time, to consider patients’ compliance, and could also include patient-reported outcome measures (PROMs) [46] to evaluate perceived esthetic, functional, and quality-of-life improvements.

## 5. Conclusions

Both ED and HPHD can be used to treat hyperdivergent class II growing patients. The two different devices can achieve comparable mandibular positional improvement. Both devices seem to preserve vertical skeletal dimensions, avoiding further mandibular clockwise rotation. Both appliances limit undesirable dentoalveolar and soft tissue effects.

## Figures and Tables

**Table 1:** Eligibility criteria for study participants.

Inclusion criteria	Male/female
	Aged between 7 and 11
	Mixed or permanent dentition
	Skeletal II class
	Hyperdivergent
	Orthodontic treatment with high-pull headgear or elastodontic device
Exclusion criteria	Evident lack of compliance in the use of the orthodontic appliance
	Genetic syndromes, cleft lip or cleft lip and palate, severe maxillary malformations, severe skeletal asymmetry
	History of speech-language therapy

**Table 2:** Cephalometric parameters.

Parameter	Definition
N	Nasion: point of maximum concavity at the level of the nasofrontal suture
S	Sella: center of sella turcica
A	Subspinal point: the innermost point of the curve joining the anterior nasal spine to the alveolar process of the upper jaw
B	Supramental point: the innermost point of the curve joining the alveolar margin of the mandible to the bony pogonion
Go	Gonion: point of construction given by the intersection of the tangent to the edge of the posterior and inferior edge of the mandible
Gn	Gnathion: most anterior and inferior point of the chin located between Pogonion and Menton
Me	Menton: lowest point of the mental symphysis contour
Ba	Basion: lower-most point of the basal pyramid of the occipital bone
Ar	Articular point: point of intersection between the posterior margin of the mandibular ramus and the inferior contour of the cranial base, representing the posterior limit of the mandibular condylar region
INI+	Upper Incisal Point: point marking the lower margin of the crown of the upper central incisor
INI−	Lower Incisal Point: point marking the upper margin of the crown of the lower central incisor
API+	Upper Apical Point: point marking the apex of the upper central incisor
API−	Lower Apical Point: point marking the apex of the lower central incisor
POG	Pogonion: the most anterior point of the mandibular symphysis, i.e., the most prominent point of the chin bone eminence
Or	Orbital: the lowest point of the lower edge of the orbit
P	Porion: the highest point of the acoustic hole
M	The most prominent point of the upper jaw bone between the alveoli of the two upper central incisors
G	Glabella: the most prominent soft tissue point of the forehead
Sn	Subnasal: the point of intersection between the upper lip and the base of the nose
POG’	Soft Tissue Pogonion: the most prominent soft tissue point of the chin
P-Or	Frankfurt plane
ANS	Anterior Nasal Spine: most prominent point of the upper jaw
PNS	Posterior Nasal Spine: the rearmost point of the hard palate
SNA	Angle determined by Sella, Nasion, and Subspinal point
SNB	Angle determined by Sella, Nasion, and Supramental point
ANB	Angle determined by Subspinal point, Nasion, and Supramental point
Wits	Distance between the orthogonal projections of points A and B on the occlusal plane
S-N	Anterior skull base plane
ANS-PNS	Bispinal plane
Gn-Me	Length of the mandibular body
Ar-Gn	Length of the mandibular branch
Go-Gn	Mandibular plane
N-Me	Total anterior facial height
S-Gn	Total posterior facial height
Occlusal plane	The line joining the distal cusp of the first molar with the cusp of the first premolar
ANS-PNS^Go-Gn	Intermaxillary angle: angle determined by the bispinal plane and mandibular plane
S-N^Go-Gn	Mandibular angle: angle determined by the plane of the skull base and mandibular plane
Occlusal-mandibular	Angle determined by occlusal plane and mandibular plane
Occlusal-palatal	Angle determined by occlusal plane and palatal plane
N-S-Ar	Angle determined by Nasion, Sella, and Articular point
S-Ar-Go	Angle determined by Sella, Articular point, and Gonion
Ar-Go-N	Angle determined by Articular point, Gonion, and Nasion
N-Go-Gn	Angle determined by Nasion, Gonion, and Gnathion
11^occlusal	Angle determined by the axis of the upper central incisor and occlusal plane
11^ANS-PNS	Angle determined by the axis of the upper central incisor and bispinal plane
41^occlusal	Angle determined by the axis of the lower central incisor and occlusal plane
41^Go-Gn	Angle determined by the axis of the lower central incisor and mandibular plane
Nasolabial	Nasolabial angle: angle determined by the base of the nose and upper lip
Facial	Facial angle: angle determined by Glabella, Subnasal point, and Soft Tissue Pogonion

**Table 3:** Demographic data of the study sample at the baseline (T0).

Group	Males (%)	Females (%)	Mean Age (SD)	CVMS 1 (%)	CVMS 2 (%)	CVMS 3 (%)
ED	11 (55%)	9 (45%)	9.2 (1.2)	14 (70%)	6 (30%)	0 (0%)
HPHD	7 (35%)	13 (65%)	9.6 (1.7)	12 (60%)	6 (30%)	2 (10%)
*p* value	0.341		0.197	0.448		

Abbreviations: ED, elastodontic device. HPHD, high-pull headgear.

**Table 4:** Descriptive statistics (mean ± standard deviation and effect size) of cephalometric parameter measurements.

	HPHD			ED		
	T0	T1	Effect Size	T0	T1	Effect Size
SNA	80.01 ± 3.24 ^A^	81.35 ± 3.76 ^A^	0.38	80.78 ± 2.66 ^A^	82.13 ± 2.77 ^A^	0.50
SNB	74.51 ± 2.89 ^A,C^	76.41 ± 2.80 ^B,D^	0.67	75.96 ± 2.13 ^A,D^	77.79 ± 2.05 ^B,C^	0.87
ANB	5.52 ± 2.14 ^A^	4.95 ± 2.06 ^A^	−0.27	4.84 ± 1.63 ^A^	4.35 ± 2.32 ^A^	−0.25
S-N^Go-Gn	39.41 ± 3.64 ^A^	37.48 ± 4.27 ^A,B^	−0.49	34.88 ± 4.83 ^A, B^	33.42 ± 4.23 ^B^	−0.32
AnsPns^Go-Gn	31.38 ± 3.61 ^A^	29.89 ± 4.76 ^A,B^	−0.36	27 ± 4.99 ^A,B^	25.6 ± 5.2 ^B^	−0.28
Occlusal–mandibular angle	18.36 ± 3.68 ^A^	17.7 ± 3.57 ^A^	−0.18	14.94 ± 4.56 ^A^	15.29 ± 3.44 ^A^	0.09
Occlusal–palatal angle	13.02 ± 2.86 ^A^	12.18 ± 2.4 ^A^	−0.32	12.08 ± 3.1 ^A^	10.3 ± 3.52 ^A^	−0.54
N-S-Ar	123.19 ± 7.51 ^A^	122.58 ± 7.2 ^A^	−0.08	123.74 ± 4.5 ^A^	123.87 ± 4.52 ^A^	0.03
S-Ar-Go	143.49 ± 8.79 ^A^	144.09 ± 7.99 ^A^	0.07	143.41 ± 4.78 ^A^	142.58 ± 5.78 ^A^	−0.16
Ar-Go-N	55.96 ± 3.18 ^A^	55 ± 3.06 ^A^	−0.31	55.24 ± 3.07 ^A^	54.62 ± 3.18 ^A^	−0.2
N-Go-Gn	76.62 ± 2.84 ^A^	75.58 ± 4.04 ^A,B^	−0.30	72.49 ± 4.27 ^A,B^	72.36 ± 3.74 ^B^	−0.03
11^Occlusal plane	55.37 ± 6.17 ^A^	55.67 ± 3.79 ^A^	0.06	58.84 ± 6.45 ^A^	59.21 ± 3.4 ^A^	0.07
11^AnsPns	111.45 ± 7.02 ^A^	112.19 ± 3.88 ^A^	0.14	109.08 ± 6.94 ^A^	110.51 ± 4.93 ^A^	0.24
41^Occlusal plane	70.04 ± 5.29 ^A^	70.05 ± 4.9 ^A^	0.00	73.13 ± 5.49 ^A^	70 ± 4.6 ^A^	−0.62
41^GoGn	91.73 ± 5.96 ^A^	92.25 ± 4.53 ^A^	0.01	91.92 ± 5.54 ^A^	94.71 ± 3.91 ^A^	0.59
Nasolabial angle	109.41 ± 9.85 ^A^	107.82 ± 8.38 ^A^	−0.17	113.63 ± 9.19 ^A^	113.96 ± 10.51 ^A^	0.03
Facial angle	159.91 ± 6.42 ^A^	159.61 ± 5.83 ^A^	−0.05	163.53 ± 4.8 ^A^	164.83 ± 5.86 ^A^	0.24

Means presenting the same uppercase letters do not show statistically significant intergroup and intragroup differences (*p* > 0.05).

## Data Availability

The data presented in this study are available upon reasonable request from the corresponding author.

## Abbreviations

HPHD, high-pull headgear; ED, elastodontic device.
